# Radiosynovectomy in Hemophilic Synovitis

**DOI:** 10.4274/Mirt.49369

**Published:** 2014-02-05

**Authors:** Zehra Özcan

**Affiliations:** 1 Ege University Faculty of Medicine, Department of Nuclear Medicine, İzmir, Turkey

**Keywords:** radioisotope therapy, Hemophilia, synovitis

## Abstract

Radisosynovectomy (RS) is a local form of radionuclide therapy used in various forms of arthritis characterized by synovitis. In hemophilic arthropathy, RS provides removal of inflamed synovium and prevents further joint damage. This review focuses on the practical aspects of radionuclide synovectomy in hemophilic patients and describes the issues both related to the methodology and post-therapeutic follow-up.

**Conflict of interest:**None declared.

## Abstract (tr)

Radyosinovektomi (RS) sinovitle seyreden artiritik hastalıklarda kullanılan lokal radyoterapi yöntemidir. Hemofilik artropatide RS tedavisi enflamasyon gösteren sinovyumun ablasyonu sağlar ve böylece eklem hasarının ilerlemesini önler. Bu makalede hemofili hastalarında RS uygulamasına ilişkin pratik noktalar ele alınmakta, uygulama şekli ve tedavi sonrası izlemle ile ilgili hususlar vurgulanmaktadır.

## INTRODUCTION

The purpose of this review is to describe the basic aspects of radionuclide synovectomy (RS) in hemophilic patients. It is aimed to provide practical advices particularly to the young Nuclear Medicine physicians who are not very familiar with intra-articular radionuclide therapy or for those recently intended to set up this method in their departments.

**The Pathogenesis of Hemophilic Synovitis**

Hemophilia is an X-chromosome linked disease caused by mutation of clotting factor genes. Intra-articular hemorrhage is the most common musculoskeletal manifestation of hemophilia which is presented by joint swelling and pain. Bleeding may occur after a minor trauma or spontaneously. The most frequently affected joint is usually knee, followed by elbow, ankles, hip and shoulder. 

In patients with severe hemophilia, the repeated episodes of joint bleeding may lead to chronic synovitis and progressive arthropathy. The pathogenesis of hemophilic synovitis is related to the accumulation of blood and blood break-down products in the joint which stimulate synovial cells (1,2). The repeated stimulations of synovial cells results in chronic inflammation and hypertrophy. Hypertrophied synovium develops a rich capillary network and creates a self-repeating cycle of hemorrhage-synovitis-hemorrhage which finally causes degenerative changes in the joint. Therefore the main therapeutic approach is to prevent articular damage which is achieved by controlling bleeding episodes, factor replacement and physiotherapy in the initial period. However, in cases with chronic hemarthrosis, open surgical or arthroscopic options can be used in addition to the non-surgical methods achieved either by chemical or radioisotopic agents. Compared to surgical synovectomy, RS is less invasive requiring minimal factor replacement prior to the procedure and could be performed as an ambulatory procedure with a relatively lower cost (3,4). The bleeding episodes and pain are significantly reduced following radiosynovectomy and improvement in range of motion is noted (5,6,7,8,9).

**Radionuclide Synovectomy**

Radionuclide synovectomy (radioisotope synovectomy, radiosynoviorthesis or radiosynovectomy) refers to the removal of the synovial membrane by intra-articular application of radioisotopes. After intra-articular administration, the radioactive colloids are phagocytized by the macrophages of the inflamed synovium. The release of beta radiation leads to coagulation necrosis, fibrosis and sclerosis of the synovial tissue also including vessels and pain receptors (3,10). If the technique is properly performed, the radioisotope affects synovial cells only due to its low range of radiation and no adverse effect is expected in the cartilage tissue, surrounding bone structures or other parts of the body. This therapeutic approach has been initially used in rheumatoid arthritis in 1950s and has been an alternative therapy in most kinds of painful arthropathies associated with chronic synovitis. In our instutition the use of RS has started in 2001 and more than 400 applications were performed in a total of 200 hemophilia patients so far.

**Patient Selection**

RS requires a multidisciplinary team work of orthopedic surgeon, radiologist, nuclear medicine physician, clinical hematologist and physiotherapist. In most hospitals, patients undergoing RS are determined by the collaborative effort of these experts and all the procedure is planned and run efficiently as a team work.

Hemophilic patients who have less than 2-3 bleeding episodes per month, non-responsive to conservative therapy and without radiologic evidence of irreversible joint destruction are considered ideal candidates of this therapy (2). In addition to the clinical evaluation of the patient, reviewing the recent X-ray films, ultrasound or MR images of the affected joint would be helpful to evaluate the synovial structure and its thickness (11). Since most patients have already some degree of articular damage before therapy, it should be noted that radionuclide therapy would not improve the joint destruction but will help to control bleeding episodes and further progression of the damage.

Absolute contraindications include pregnancy, lactation, the presence of ruptured Baker’s cyst, massive hemarthrosis and septic local infections. The presence of joint instability and severe cartilage destruction are considered as relative contraindications (12). In young children, RS should be used when the clinical benefit is likely to outweigh the potential risks.

**Preparations Prior to the Procedure**

The steps of this therapeutic procedure are slightly different than the other therapeutic applications due to the use radioactive tracers. In most centers, all patients who are planned to receive RS are scheduled consecutively according to the delivery of the isotope. So the human and radioactive resources are utilized in a more efficient way. The selection of radioisotope and administration dose is determined by the Nuclear Medicine physician considering the age, body height and weight, and the joint that will be injected.

Radionuclide therapy should also be planned according to the national regulations of therapeutic isotopic applications. Due to the radiation safety concerns for patients and the medical staff, the injection room, monitoring of radioactive substances, the disposal of radioactive wastes and all the other related safety precautions should be managed by the Nuclear Medicine physician and the physicist. The measurement of injected dose, the use of finger dosimeters, acrylic syringe protectors or nitrile or vinyl gloves should be considered in terms of safety precautions (10).

It should also be noted that hemophilic patients will require factor replacement and other supportive measures prior to the radionuclide administration, therefore close collaboration with the hematologist is essential.

Patients and their families should be informed about the details of the procedure and written consent should be obtained. Informed consent should include information regarding the whole procedure, therapeutic effect and potential complications (12).

**Types of Radioisotopes and Their Properties**

There are several radioisotopes that can be used for radionuclide synovectomy. The ideal radioisotope should be a pure beta emitter, with a limited range of penetration through the tissues and a moderate physical half-life. In order to achieve optimal synovial distribution, the radiopharmaceutical should be in the colloidal form. The size of the colloid should be small enough to be phagocytized and large enough to avoid extra-articular escape. A particle size of at least 5-10 nm is essential in order to avoid leakage. In a large joint like knee, Y-90 is preferred due to its longer range in tissue. In medium sized joints, Re-186 is chosen. P-32 which has been frequently used in the past, is no more used in EU for synovectomy but available in North America and some other countries. The most frequently used isotopes are listed in Table 1.

**The Technique**

**Figure 1:**
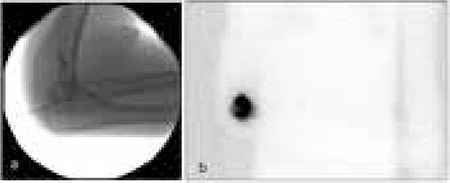
Following insertion of the needle to the elbow, its position in the joint is checked on a spot view (a). After the injection of the Re-186 colloidal sulphide, gamma image (b) of the same patient shows intra-ar- ticular activity accumulation. No evidence of leakage outside the elbow is noted.

When the facility is arranged properly and radiation safety precautions are taken, intra-articular injection of the radiocolloid is performed by an experienced physician under strict sterile conditions. The use local anesthesia is advised. Apart from the knee, all joints are usually punctured by fluoroscopy to confirm the intra-articular position of the needle prior to the isotope administration (Figure 1a). Once the needle has been advanced into the joint, synovial fluid is aspirated before the radiocolloid injection so that the correct position of the needle is ensured. In cases with joint effusion, synovial fluid is aspirated. In order to avoid radionuclide contamination through the needle tract, it should be flushed before and during the withdrawal with 0.9% saline.

At the end of the procedure several light flexion and extension movements are performed to have an optimum distribution of the tracer in the joint space. Soon after the injection, joint is immobilized (splinting) for at least 48 hrs. to reduce the transport of particles through the lymphatic system.

If necessary, two joints can be injected at the same session, such as a large and medium sized joint. Some experts suggest the use of simultaneous local corticosteroid administration of during the radiosynovectomy to reduce acute synovitis and to achieve a better therapeutic effect, however this issue is not universally accepted (4,12).

Post-therapy imaging should be recorded in order to confirm radiocolloidal distribution in the injected joint. Bremsstrahlung and standard gamma imaging can be performed after Y-90 and Re-186 colloid injections respectively (Figure 1b).

**Therapeutic Effect and Follow-up**

Soon after radiosynovectomy, slight increase in pain and swelling may occur due to the temporar synovial reaction to the radioisotopes. Local application of cold and analgesics are helpful for the pain relief. Actual therapeutic effect typically occurs 1 week after intra-articular injection but sometimes may extend to 2-3 weeks. Successful response is noted usually in 60 to 80% of the patients. Patients with mild joint disease are likely to have the greatest long-term preservation of joint function.

If there is no response by 6 weeks, it indicates failure to therapy. The minimum interval between 2 consecutive injections is 6 months. In case of 2 unresponsive interventions, no further radiosynovectomy is attempted (12).

**Precautions After the Procedure**

Soon after radiosynovectomy, the joint should be immobilized for at least for 48 hrs. The patients should be noticed about avoiding severe exercise and weight bearing on that side which may cause extra-articular leakage and decreases therapeutic effect.

Radioactive urinary excretion may be highest during the first 2 days following therapy therefore rigorous hygiene, twice flushing of the toilet is recommended. In incontinent patients bladder catheterization is suggested prior to the radiosynovectomy. In women, pregnancy should be avoided at least for 4 months (12). Except these points, patients may continue their usual daily lives.

Although very rare, patients should be informed about the potential local adverse reactions such as ulceration or radionecrosis in the injection site. These are usually related to the improper injection technique and radiocolloid leakage.

**Long Term Radiation Safety Considerations**

While radiosynovectomy appears to be as a safe and simple therapeutic option in hemophilic synovitis, it is known that exposure to ionizing radiation may potentially induce chromosomal aberrations particularly in young children. Recently some concerns are raised after the publication of 2 children developing acute lymphoblastic leukemia (ALL) within 1 year following radiosynovectomy (13,14). However, due to the short interval between radiation exposure and malignancy, the causal relationship between P-32 and development of leukemia cannot be established exclusively in these patients. Moreover, both children had already additional autoimmune disorders (15,16).

Regarding the chromosomal changes after RS, several authors have shown that genotoxic radiation doses delivered to the peripheral lymphocytes are not statistically significant (17,18,19,20,21). However, transient increase in micronuclei of peripheral lymphocytes may be expected soon after Y-90 therapy which disappeared at 90^th^. day (20).

While the current literature is limited about the long-term risks of cancer, a recent retrospective study including 2412 adult patients treated with RS revealed no increase in the risk of cancer compared to the general population (22). This was in agreement with the study of Vuorela et al (23). In their study comparing the medical records of 1228 rheumatoid arthritis patients with and without RS, no evidence of increase in cancer incidence ratio was found following Y-90 therapy.

In conclusion, the current literature shows that radisoynovectomy is a safe and efficient therapy in patients with hemophilic synovitis. However, close collaboration of physicians guiding the therapy and careful application of radiosynovectomy are essential for optimal medical care both in the short and the long-term follow-up of these patients.

**Conflicts of interest:** None. 

## Figures and Tables

**Table 1:**
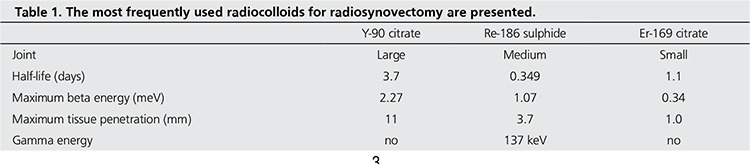
The most frequently used radiocolloids for radiosynovectomy are presented.
